# Linear and exponential TAIL-PCR: a method for efficient and quick amplification of flanking sequences adjacent to Tn5 transposon insertion sites

**DOI:** 10.1186/s13568-017-0495-x

**Published:** 2017-11-02

**Authors:** Xianbo Jia, Xinjian Lin, Jichen Chen

**Affiliations:** 1 Institute of Soil and Fertilizer, Fujian Academy of Agricultural and Sciences, Wusi Road, Fuzhou, 350001 Fujian People’s Republic of China; 2 Institute of Applied Ecology, Fujian Agriculture and Forestry University, Fuzhou, People’s Republic of China

**Keywords:** LETAIL-PCR, Genome walking, Linear amplification, Tn5 transposon

## Abstract

Current genome walking methods are very time consuming, and many produce non-specific amplification products. To amplify the flanking sequences that are adjacent to Tn5 transposon insertion sites in *Serratia marcescens* FZSF02, we developed a genome walking method based on TAIL-PCR. This PCR method added a 20-cycle linear amplification step before the exponential amplification step to increase the concentration of the target sequences. Products of the linear amplification and the exponential amplification were diluted 100-fold to decrease the concentration of the templates that cause non-specific amplification. Fast DNA polymerase with a high extension speed was used in this method, and an amplification program was used to rapidly amplify long specific sequences. With this linear and exponential TAIL-PCR (LETAIL-PCR), we successfully obtained products larger than 2 kb from Tn5 transposon insertion mutant strains within 3 h. This method can be widely used in genome walking studies to amplify unknown sequences that are adjacent to known sequences.

**Electronic supplementary material:**

The online version of this article (10.1186/s13568-017-0495-x) contains supplementary material, which is available to authorized users.

## Introduction

Tn transposons are widely used to study functional genes in bacteria by insertion knockouts (Reznikoff 2008). Amplification of flanking regions adjacent to the insertion sites is necessary for the detection of mutant genes generated by random Tn transposon insertion. However, the flanking sequences adjacent to the known sequences cannot be amplified by conventional PCR because the sequences are unknown; therefore, specific primers pairs cannot be designed to amplify such regions. To solve this issue, currently, the random primed method and the ligation-mediated method are the most commonly used methods for flanking sequence amplification (Uchiyama and Watanabe 2006; Yan et al. 2003). The random primed method is based on primer design and the optimization of the PCR protocol. For example, Liu and Whittier (1995) developed a version of random primed PCR termed thermal asymmetric interlaced PCR (TAIL-PCR), which employs three rounds of PCR with three specific (SP) primers that have a high T_m_ from the known sequences and four arbitrary degenerate (AD) primers that have a low T_m_ for binding to the unknown sequences. This method utilizes the specificity of PCR through increasing the binding efficiency of SP primers and decreasing the binding efficiency of AD primers at higher annealing temperatures. Subsequently, Liu and Chen (2007) improved the efficiency of TAIL-PCR and developed a new procedure termed high-efficiency thermal asymmetric interlaced PCR (hiTAIL-PCR). This procedure combines the advantages of TAIL-cycling and suppression-PCR, thus significantly improving the amplification efficiency of the target flanking sequences. However, non-specific products are also often amplified when using the random primed methods. Completing three or more rounds of PCR is also time consuming. Ligation-mediated methods are based on digesting sequences with restriction enzymes, ligating sequences into a plasmid, transforming bacteria with plasmids and screening for strains containing the flanking regions. For example, to identify the flanking sequences of T-DNA insertions in plants, (Cottage et al. 2001) digested plant genomic DNA with blunt-cutting restriction enzymes and annealed an adapter sequence to the blunt-ended unknown region. Then, specific primers for the T-DNA and specific primers for the adaptor sequence were used to amplify the unknown flanking sequences using a conventional PCR procedure. A genome walking procedure termed loop-linker PCR was developed by adding a loop-linker adapter to the unknown region digested with restriction enzymes, which enhances amplification efficiency by suppressing the generation of nonspecific PCR products (Trinh et al. 2012). Several other ligation-mediated methods have also been reported (Yin and Largaespad 2007; Rosenthal and Jones 1990; Huang et al. 2000; Tsaftaris et al. 2010), these Ligation-mediated methods are effective and highly specific, but they require restriction enzyme digestion, ligation, DNA purification and cloning, which are laborious and time-consuming and are not suitable for high-throughput use, such as screening a mutant library with hundreds of clones. Random primed methods rely on PCR protocols, which are faster and simpler than ligation-mediated methods. However, these random primed methods produce non-specific amplification. Additionally, to amplify long flanking sequences, the extension time of the PCR procedure in these methods is usually more than 3 min/cycle, resulting in these methods taking more than 1 day to complete, even for products < 1.5 kb (Liu and Chen 2007). So, a method that is fast, efficient and specific has yet to be considered.

On the basis of comparing the advantages and disadvantages of the methods mentioned above, here, we present a more effective method termed LETAIL-PCR to obtain the flanking sequences adjacent to Tn5 transposon insertion sites. On the basis of hiTAIL-PCR, linear pre-amplification combined with high-fold dilution of the pre-amplification product was used to decrease non-specific amplification. A version of high speed DNA polymerase was used to shorten the amplification time. Using this method, long PCR products can be obtained with high specificity in a short time.

## Materials and methods

### Strains and DNA template preparation

*Serratia marcescens* FZSF02 was isolated from soil in Fuzhou, China and was deposited in China General Microbiological Culture Collection Center (CGMCC) with the strain number CGMCC 1.16177. Tn5 mutant strains of *Serratia marcescens* FZSF02 were obtained using the EZ-Tn5™ <KAN-2>Tnp Transposome™ Kit (Epicentre, USA). DNA templates were prepared with a Bacteria Genomic DNA Kit (TIANGEN, China) by extracting the genomes of wild-type FZSF02 and mutant FZSF02 strains. Three DNA samples from three FZSF02 mutant strains were used in this study. Flanking sequences adjacent to Tn5 transposon insertion sites of these three samples were submitted to GenBank with the accession numbers of MF034039 (sample 1), MF034040 (sample 2) and MF034041 (sample 3).

### Primers and reagents

The non-specific primers LAD1 and LAD3 and the specific primer AC1 were designed by Liu and Chen (2007). The primers F389, F536 and F772 were designed from the known region of the insertion site (Additional file 1). All primers are shown in Table 1.

**Table 1:** Primers used in this method

Primer name	Sequence	T_m_ (°C)	Description
F389	5′-TCAAGCATTTTATCCGTACTCCTG-3′	55.39	Designed by our lab
F536	5′-CGGTTGCATTCGATTCCTGTTTGTA-3′	58.73	Designed by our lab
F772	5′-TAGGTTGTATTGATGTTGGACGAG-3′	55.09	Designed by our lab
LAD1	5′-ACGATGGACTCCAGAG(G/C/A)N(G/C/A)NNNGGAA-3′		(Liu and Chen 2007)
LAD3	5′-ACGATGGACTCCAGAG(T/A/C)N(A/G/C)NNNCCAC-3′		(Liu and Chen 2007)
AC1	5′-ACGATGGACTCCAGAG-3′	49.15	(Liu and Chen 2007)

### Overview of the method

This method is divided into three steps. The first step includes two reactions:a linear reaction and an exponential reaction. For the linear reaction, a total PCR reaction volume of 20 μL was prepared containing 10 μL of prime STAR Max DNA polymerase (TAKARA Japan), 5 μL of primer F389 (100 pmol), 1 μL of genomic DNA (about 100 ng) and 4 μL of ddH_2_O. The thermal conditions of the linear reaction are shown in Table 2. The product was diluted 100-fold and used as the template for the subsequent exponential reaction. For the exponential reaction, a total PCR reaction volume of 20 μL was prepared containing 10 μL of prime STAR Max DNA polymerase (TAKARA Japan), 1 μL of primer F389 (20 pmol), 0.5 μL of primer LAD1 (10 pmol), 0.5 μL of LAD3 (10 pmol), 1 μL of template and 7 μL of ddH_2_O. The reaction conditions for this exponential reaction are shown in Table 2. The PCR product was diluted 100-fold and used as the template for the second step. The second step also included two reactions: a linear reaction and an exponential reaction. For the linear reaction, a total PCR reaction volume of 20 μL was prepared containing 10 μL of prime STAR Max DNA polymerase (TAKARA Japan), 5 μL of primer F536 (100 pmol), 1 μL of the diluted PCR product from the first step as the template and 4 μL of ddH_2_O. The conditions of this linear reaction are shown in Table 2. The product was diluted tenfold and used as the template for the following exponential reaction. For the exponential reaction, a total PCR reaction volume of 20 μL was prepared containing 10 μL of prime STAR Max DNA polymerase (TAKARA Japan), 2 μL of primer F536 (40 pmol), 2 μL of primer LAC1 (40 pmol), 1 μL of the diluted PCR product from the linear reaction as the template and 7 μL of ddH_2_O. The reaction conditions of this exponential reaction are shown in Table 2. The PCR product was diluted ~ 10- to 50-fold for use as the template of the next third step. The third step contains one exponential reaction. For this reaction, a total PCR reaction volume of 20 μL was prepared containing 10 μL of prime STAR Max DNA polymerase (TAKARA Japan), 1 μL of primer F772 (20 pmol), 1 μL of primer LAC1 (20 pmol), 1 μL of the diluted PCR product from the second step as the template and 7 μL of ddH_2_O. The reaction conditions of this exponential reaction are shown in Table 2.

**Table 2:** Thermal conditions used in this method

Steps	Amplification style	Thermal condition	Dilution (fold)
1	Linear	98 °C 10 s, 62 °C 5 s, 72 °C 30 s; 20 cycles	100
	Exponential	98 °C 10 s, 25 °C 5 s, 72 °C 30 s; 1 cycle 98 °C 10 s, 58 °C 5 s, 72 °C 30 s; 18 cycles	100
2	Linear	98 °C 10 s, 62 °C 5 s, 72 °C 30 s; 20 cycles	10
	Exponential	98 °C 10 s, 68 °C 5 s, 72 °C 30 s, 98 °C 10 s, 63 °C 5 s, 72 °C 30 s, 98 °C 10 s, 50 °C 5 s, 72 °C 30 s; 7 cycles	100
3	Exponential	98 °C 10 s, 68 °C 5 s, 72 °C 30 s, 98 °C 10 s, 63 °C 5 s, 72 °C 30 s, 98 °C 10 s, 50 °C 5 s, 72 °C 30 s; 13 cycles	

### Sequence analysis

PCR products from the three exponential amplifications were electrophoresed on 1% agarose gels and visualized after ethidium bromide staining. The DNA bands on the gels were purified with a Gel Extraction Kit (OMEGA USA) and were sequenced directly with the Sanger method. Chromas 2.22 was used to evaluate the sequence quality by examining the chromatograms.

## Results

### Amplification of the flanking unknown sequence

**Fig. 1:**
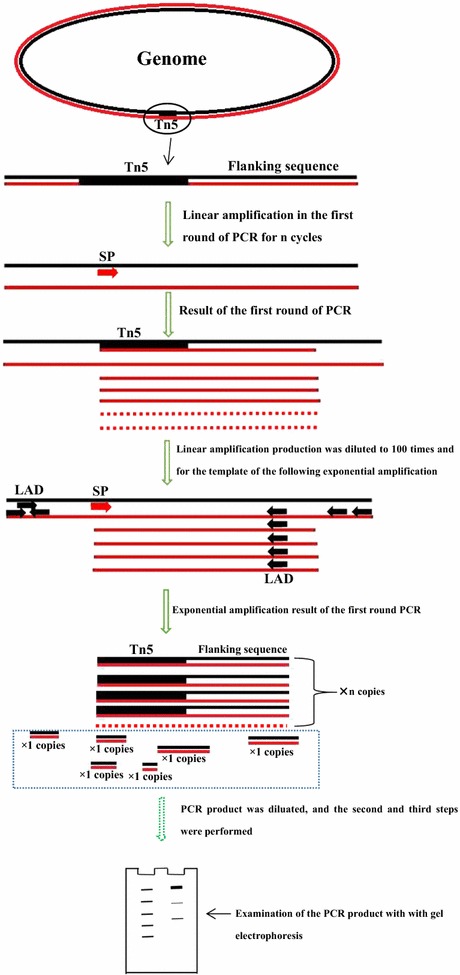
The genome walking method scheme. Black lines represent the sense strand of the DNA, and red lines represent the anti-sense strand. Black shadow positions on the DNA represent the known sequence, and the residual white parts on the DNA represent unknown sequences. Red arrows (SP) represent specific primers. Black arrows (LAD) represent degenerate primers

**Fig. 2:**
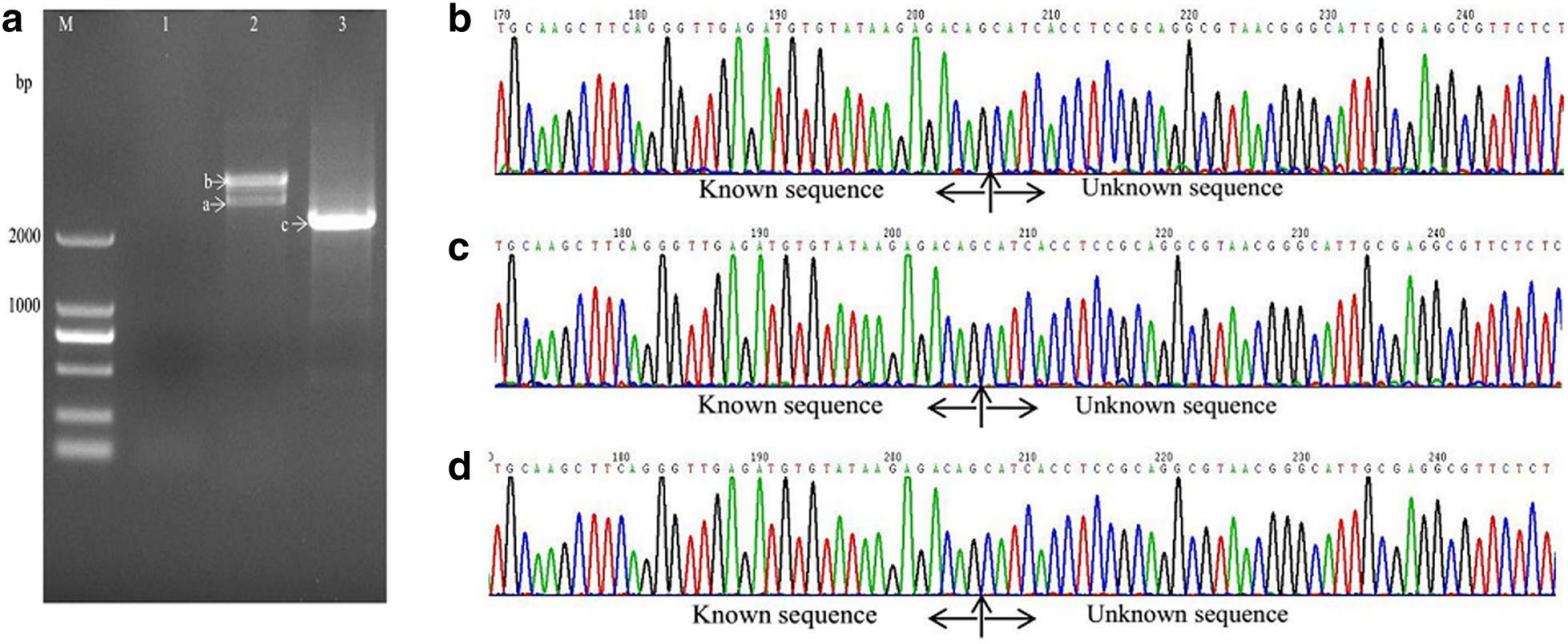
Amplification and sequencing results of the sequence flanking the Tn5 insertion site of sample 1. **a** Amplification products shown on an agarose gel. Lanes 1–3 show the results of steps 1–3, respectively. **b** Chromatograms of DNA in band a. **c** Chromatograms of DNA in band b. **d** Chromatograms of DNA in band c

**Fig. 3:**
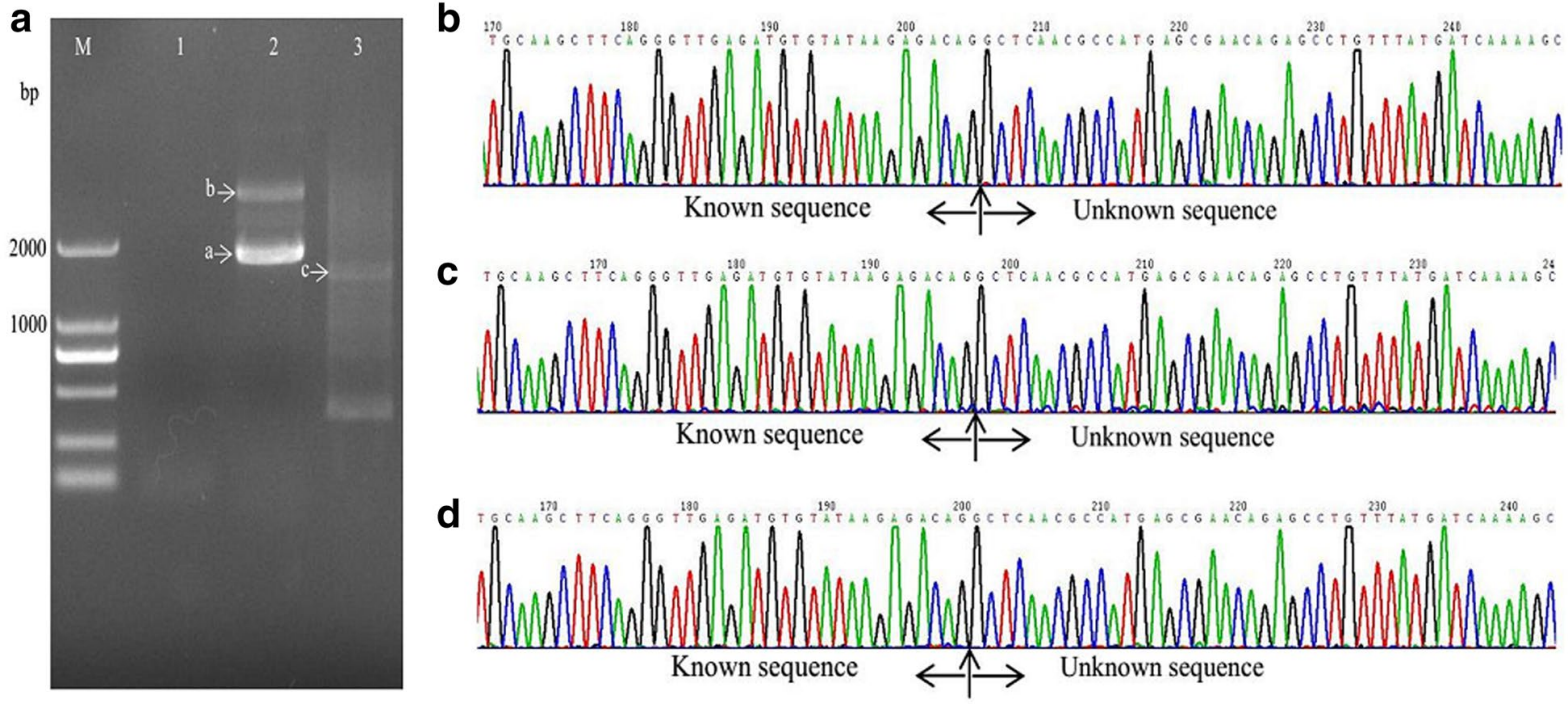
Amplification and sequencing results of the sequence flanking the Tn5 insertion site of sample 2. **a** Amplification product shown on an agarose gel. Lanes 1–3 show the results of steps 1–3, respectively. **b** Chromatograms of DNA in band a. **c** Chromatograms of DNA in band b. **d** Chromatograms of DNA in band c

**Fig. 4:**
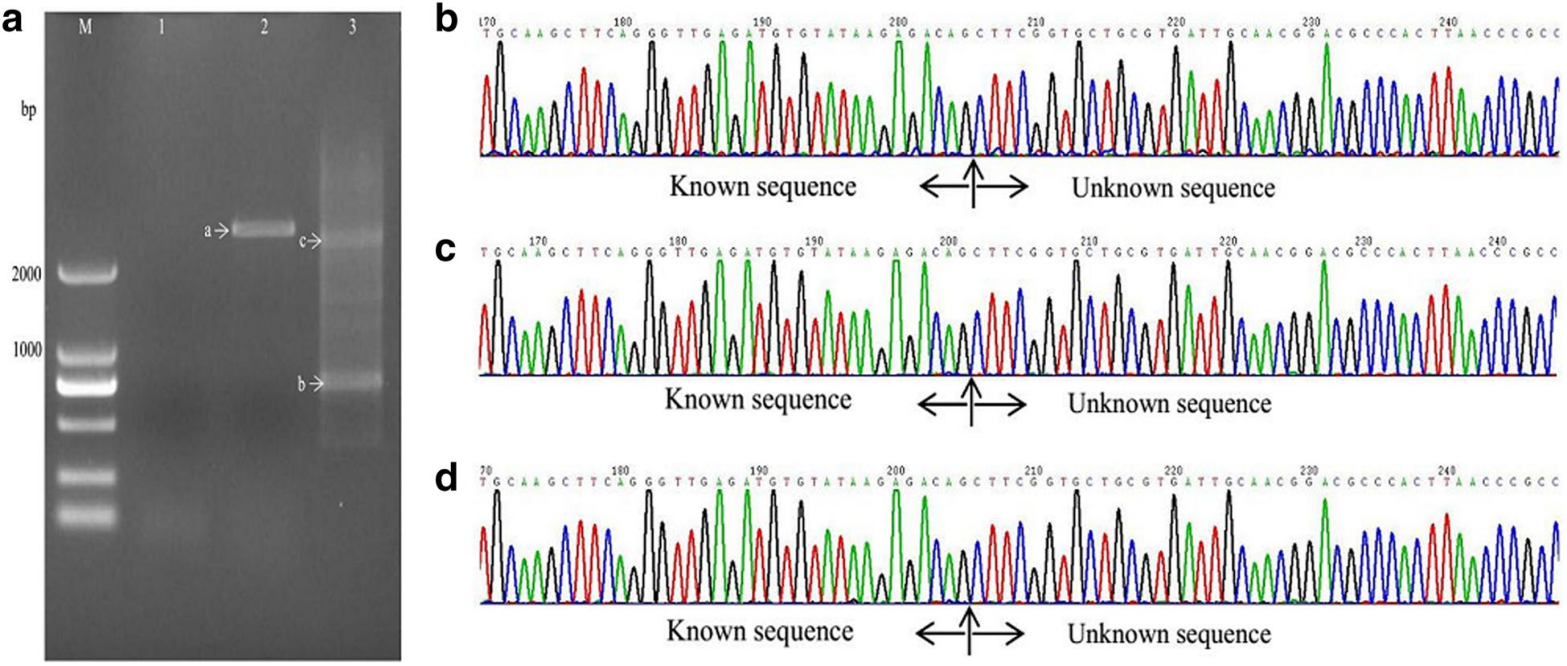
Amplification and sequencing results of the sequence flanking the Tn5 insertion site of sample 3. **a** Amplification product showed on agarose gel. Lanes 1–3 show the results of steps 1–3, respectively. **b** Chromatograms of DNA in band a. **c** Chromatograms of DNA in band b. **d** Chromatograms of DNA in band c

**Fig. 5:**
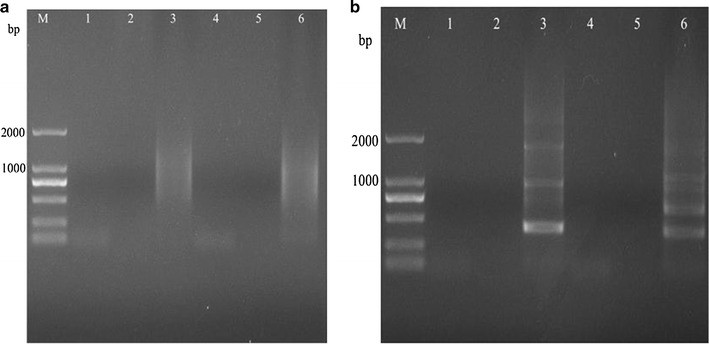
Amplification results of a non-Tn5 insertion sample and non-linear amplification of sample 1. **a** Amplification of a non-Tn5 insertion sample with the method described in this study. Each step was repeated twice. Lanes 1 and 4 show the results of the first step. Lanes 2 and 5 show the results of the second step. Lanes 3 and 6 show the results of the third step. **b** Amplification results of sample 1 without linear amplification. Each step was repeated twice. Lanes 1 and 4 show the results of the first step. Lanes 2 and 5 show the results of the second step. Lanes 3 and 6 show the results of the third step

With the method described in this study (Fig. 1, Table 2), samples from all three mutant strains showed one or two visible bands larger than 2 kb (Figs. 2a, 3, 4a) after the second exponential amplification step. After the third step, the results varied significantly between the three samples. A distinct band that was slightly larger than 2 kb appeared in sample 1 (lane 3 of Fig. 2a), a weak band of approximately 1.5 kb appeared in sample 2 (lane 3 of Fig. 3a), and a weak band larger than 2 kb and a weak band of approximately 750 bp appeared in sample 3 (lane 3 of Fig. 4a). There were no visible bands in the three lanes of the gels for the DNA sample of the wild-type strain without Tn5 transposon (Fig. 5a). A non-linear amplification control was carried out with sample 1, and the result shows only lane 3 and lane 6 (Fig. 5b), which correspond to the third amplification step, have dispersion zones.

### Sequencing of the amplified DNA bands

The visible DNA bands on the gels of sample 1, sample 2 and sample 3 were sequenced, and the chromatograms showed that these band all contained target sequences adjacent to the inserted sequences (Figs. 2b–d, 3b–d, 4b–d). Moreover, the chromatograms showed almost no miscellaneous peaks, demonstrating that the DNA bands were very pure. Detailed DNA sequences of these DNA bands are shown in Additional file 1.

## Discussion

The principle basis of LETAIL-PCR is outlined in Fig. 1. In detail, linear amplifications with a specific primer were carried out before the exponential amplifications to increase the relative quantity of the target sequences. For the first step, the linear amplification product was diluted 100-fold to decrease the relative genomic DNA concentration. Therefore, after the linear amplification and dilution, non-specific amplification in the exponential amplification with the specific primer F389 and degenerate LAD primers is significantly decreased. For the second step, the product of the first step was diluted 100-fold for use as the template, linear amplification with only the specific primer F536 was carried out, and the amplification product was diluted tenfold for use as the template for the following exponential amplification. After the two linear amplifications and the two exponential amplifications described above were complete, the specific amplification was increased. There are several important minor points for the linear amplification. First, the primer concentration was relatively high (100 pmol/20 μL reaction mixture) to enhance the specific annealing of the primer with the target sequence. Second, the annealing temperature of the linear amplification was set at a higher temperature of 62 °C to reduce the possibility of non-specific amplification. Third, the exponential amplifications of the first and second steps were both set at ~ 18–21 cycles, rather than the normal ~ 30 cycles, to decrease the generation of non-specific products. Fourth, the 100-fold dilution after the linear amplification in the first step decreases the genomic DNA concentration to avoid non-specific amplification of the LAD1 and LAD3 primers with genomic DNA. The 100-fold dilution of the first exponential amplification product before the linear amplification of the second step decreases the concentration of LAD1 and LAD3 primers to avoid non-specific amplification in this linear amplification. These minor points all ensured the specificity of the amplification.

This LETAIL-PCR method also adopted the reported TAIL-PCR method (Liu and Chen 2007). TAIL-PCR uses specific primers from the known sequence (F389, F536 and F772 in this study) with T_m_ values higher than that of the primer for the unknown sequence (LAC1). Therefore, in the PCR process, the target sequence can be amplified with higher annealing temperatures. Compared with the previous reports, linear amplification before exponential amplification further increased the specificity of TAIL-PCR. In our study, PCR products showed more than one band on the agarose gels (Figs. 2a, 3a, 4a), but all bands contained target sequences adjacent to the Tn5 transposon insertion sites. Specific products were found in the second step, which is accordance with the description of a similar protocol in a previous report (Luo et al. 2011).

In traditional random primed based methods, DNA polymerases have a theoretical extension speed of approximately 1 kb/min. The extension time required for every cycle in these PCR procedures is more than 3 min to obtain long PCR products, which is very time consuming. In this study, to obtain longer unknown sequences, amplifying long enough single stranded DNA by linear amplification is necessary. So those faster DNA polymerases which can amplify longer DNA sequences in much shorter time are more suitable than the common Taq DNA polymerases. In this presented method, a type of fast DNA polymerase, PrimeSTAR Max DNA Polymerase (Takara, Japan), with a high extension speed of 5 s/kb was used. The PCR program for the method with this kind of DNA polymerase decreased the time requirement to 3 h compared with approximately 7 h for other genome walking methods (Liu and Chen 2007; Li et al. 2015). Though significantly less time was spent using this DNA polymerase and PCR program, the obtained PCR products were more than 2 kb, which are much longer than those in many previous reports (Singer and Burk 2003; Schmidt et al. 2007; Zhang et al. 2011; Ma et al. 2014; Reddy et al. 2008; Xu et al. 2013).

In conclusion, a modified LETAIL-PCR method was developed to amplify the flanking sequence of the Tn5 transposon insertion sites. Two linear amplifications combined with high-fold dilution can significantly enhance the specificity of the amplification. The DNA polymerase and the corresponding amplification program allowed larger PCR products to be obtained in a short amount of time using this method. The high efficiency and short time required make this method very appealing for genome walking studies, including Tn transposon insertion sequence in microbes and flanking sequence identification for T-DNA insertion in plants.

## Additional file

**Additional file 1.** 1. Insertion Tn5 sequence and the positions of the specific primers. 2. Sequencing results of the three samples.

## Abbreviation

TAIL-PCR: thermal asymmetric interlaced polymerase chain reaction
