# Supplementary material for: Linear and exponential TAIL-PCR: a method for efficient and quick amplification of flanking sequences adjacent to Tn5 transposon insertion sites
Source: AMB Express. 2017 Nov 2;7:195. doi: 10.1186/s13568-017-0495-x (PMC5668224; doi:10.1186/s13568-017-0495-x)
Supplement: Supplementary file 1 — Additional file 1. 1. Insertion Tn5 sequence and the positions of the specific primers. 2. Sequencing results of the three samples. [file 13568_2017_495_MOESM1_ESM.docx]

**Additional file**

**Linear and exponential TAIL-PCR: a method for efficient and quick amplification of flanking sequences adjacent to Tn5 transposon insertion sites**

Xianbo Jia^1,2^, Xinjian Lin^1^ and Jichen Chen^1*^

^1^ Institute of Soil and Fertilizer, Fujian Academy of Agricultural and Sciences, Fuzhou, People’s Republic of China

^2^ Institute of Applied Ecology, Fujian Agriculture and Forestry University, Fuzhou, People’s Republic of China

**1. Insertion Tn5 sequence**

CTGTCTCTTATACACATCTCAACCATCATCGATGAATTGTGTCTCAAAATCTCTGATGTTACATTGCACAAGATAAAAATATATCATCATGAACAATAAAACTGTCTGCTTACATAAACAGTAATACAAGGGGTGTTATGAGCCATATTCAACGGGAAACGTCTTGCTCGAGGCCGCGATTAAATTCCAACATGGATGCTGATTTATATGGGTATAAATGGGCTCGCGATAATGTCGGGCAATCAGGTGCGACAATCTATCGATTGTATGGGAAGCCCGATGCGCCAGAGTTGTTTCTGAAACATGGCAAAGGTAGCGTTGCCAATGATGTTACAGATGAGATGGTCAGACTAA

**F389**

ACTGGCTGACGGAATTTATGCCTCTTCCGACCATCAAGCATTTTATCCGTACTCCTGATGATGCATGGTTACTCACCACTGCGATCCCCGGAAAAACAGCATTCCAGGTATTAGAAGA

**F536**

TATCCTGATTCAGGTGAAAATATTGTTGATGCGCTGGCAGTGTTCCTGCGCCGGTTGCATTCGATTCCTGTTTGTAATTGTCCTTTTAACAGCGATCGCGTATTTCGTCTCGCTCAGGCGCAATCACGAATGAATAACGGTTTGGTTGATGCGAGTGATTTTGATGACGAGCGTAATGGCTGGCCTGTTGAACAAGTCTGGAAAGAAATGCATAAACTTTTGCCATTCTCACCGGATTCAGTCGTCACTCATGGTGATTTCTCACTTGATAACCTTATTTTTGACGAGGGGAAATT

**F772**

AATAGGTTGTATTGATGTTGGACGAGTCGGAATCGCAGACCGATACCAGGATCTTGCCATCCTATGGAACTGCCTCGGTGAGTTTTCTCCTTCATTACAGAAACGGCTTTTTCAAAAATATGGTATTGATAATCCTGATATGAATAAATTGCAGTTTCATTTGATGCTCGATGAGTTTTTCTAATCAGAATTGGTTAATTGGTTGTAACACTGGCAGAGCATTACGCTGACTTGACGGGACGGCGGCTTTGTTGAATAAATCGAACTTTTGCTGAGTTGAAGGATCAGATCACGCATCTTCCCGACAACGCAGACCGTTCCGTGGCAAAGCAAAAGTTCAAAATCACCAACTGGTCCACCTACAACAAAGCTCTCATCAACCGTGGCGGGGATCCTCTAGAGTCGACCTGCAGGCATGCAAGCTTCAGGGTTGAGATGTGTATAAGAGACAG

**2. Sequencing results of the three samples** (Sequences of red color were known sequence of Tn5; sequences of black color are unknown sequences flanking Tn5 sequence)

**Sequencing result of sample1 lane(a)**

CCAACCGGGCCTTTATCGACTTTTGCTGAGTTGAGGATCAGATCACGCATCTTCCCGACAACGCAGACCGTTCCGTGGCAAAGCAAAAGTTCAAAATCACCAACTGGTCCACCTACAACAAAGCTCTCATCAACCGTGGCGGGGATCCTCTAGAGTCGACCTGCAGGCATGCAAGCTTCAGGGTTGAGATGTGTATAAGAGACAGCATCACCTCCGCAGGCGTAACGGGCATTGCGAGGCGTTCTCTCACCGCTTTGGCGGCGTTCAGCCCGGCTAACACCGCGCTTTCGATATTACCGATGCCCACGCTGTCCTGATTGGCGAAGTGAATATTCTCAAACGGCATGCTGGCCTGGTGAAACACGCCCTGAGCCTGTTGTCCGGCGACCGAGGTCACCAGACCCGCGGGCCAGAACCAGATTTGAATGTCTTCAATGATGTCGGCGTCAATGCCCATGTTGCCCAGCACTTTGGCGATTTCGACATAGGTCTGTTGCTGCAGCGCCGGGAACTCGTTCTGCGCGGTGCGACCTTGCCGCTCTGGGCGAGTGGTCGGTTTGAGCAGGGTTAATACCCCGGTTTCCGCGCTGAGGTGATTGTCCATCCAATTGGCGATCAGGCAGGTGCCGGCTTTGCTCCAATAGTAAGGTGTGCGGGTGTTTTCCGGCTCGATCAGATAGCCGCCGAACGAGTGTTCGAGGATCGGTTTTTTCAACAGCACGTTCGCCAGGTAGTAATCTTCATGGCGGATCTGACGGATCGCCGCCAATTGCTCTTTGGGCAGGGCGGGCAACCATGTGGGCACATGCGTTTTGGGGGCTGCCCAGATCACTTGCTTGGCTCTGATTTCGCTGGCCGCGCCATTGACGCTGAACCGGCATCACCGTTTCGTCGCCGCCGGGCGTGATGCTTTGAATCTGTACGCGGCTCTGCAGCCTGCACGTCGTTTTTGCTCCTCAGATATTCGAGTAGCCCGGCGCTGATGCTGCCGTTAACGTCAGGCAGCGTGACAGATTGCTGCGTAATACCTCACAGGAAGTGTCATCCGAACTTAGGCGGAGATTCGCGAAACTGAGACATTCCGGCGCACGTTGTGCTGACGCATTCTCAACACCTGCCGAATTTCTCGTGGCCACAATCGC

**Sequencing result of sample1 Lane(b)**

AGGGGGGGGGGTTGTATCGACTTTTGCTGAGTTGAGGATCAGATCACGCATCTTCCCGACAACGCAGACCGTTCCGTGGCAAAGCAAAAGTTCAAAATCACCAACTGGTCCACCTACAACAAAGCTCTCATCAACCGTGGCGGGGATCCTCTAGAGTCGACCTGCAGGCATGCAAGCTTCAGGGTTGAGATGTGTATAAGAGACAGCATCACCTCCGCAGGCGTAACGGGCATTGCGAGGCGTTCTCTCACCGCTTTGGCGGCGTTCAGCCCGGCTAACACCGCGCTTTCGATATTACCGATGCCCACGCTGTCCTGATTGGCGAAGTGAATATTCTCAAACGGCATGCTGGCCTGGTGAAACACGCCCTGAGCCTGTTGTCCGGCGACCGAGGTCACCAGACCCGCGGGCCAGAACCAGATTTGAATGTCTTCAATGATGTCGGCGTCAATGCCCATGTTGCCCAGCACTTTGGCGATTTCGACATAGGTCTGTTGCTGCAGCGCCGGGAACTCGTTCTGCGCGGTGCGACCTTGCCGCTCTGGGCGAGTGGTCGGTTTGAGCAGGGTTAATACCCCGGTTTCCGCGCTGAGGTGATTGTCCATCCAATTGGCGATCAGGCAGGTGCCGGCTTTGCTCCAATAGTAAGGTGTGCGGGTGTTTTCCGGCTCGATCAGATAGCCGCCGAACGAGTGTTCGAGGATCGGTTTTTTCAACAGCACGTTCGCCAGGTAGTAATCTTCATGGCGGATCTGACGGATCGCCGCCAATTGCTCTTTGGGCAGGGTGGGCAACCAGGTGGGCACATGCGTTTTGGGGGCTGCCCAGATCACTTGTTTGGCTCTGATTTCGCTGGCCGCGCCATTGACGCTGAACCGGATCACCGTTTCGTCGCCGCCGGGCGTGATGCTTTGAAGCTGTACGCGGCTTTGCAGCCTGACGTTGTTTTGCTCCTCAGATAGTCGAGCAGCCCGGCGCTGATGCTGCCGTTACCGCCCGGCAGCGTGACCAGATTGCCGCGTAAATACCCCCACCAGGAAGTGCAATCGACAAAGCGAGACTTCGCGAATATCGAGACATTCCACGCCGCAGCGTGTGCTGACGGCATTCTCCTACCAGCTTGCCGAT

**Sequencing result of sample1 Lane(c)**

CTTCTCCGGCGCCTTTTCGACTTTTGCTGAGTTGAGGATCAGATCACGCATCTTCCCGACAACGCAGACCGTTCCGTGGCAAAGCAAAAGTTCAAAATCACCAACTGGTCCACCTACAACAAAGCTCTCATCAACCGTGGCGGGGATCCTCTAGAGTCGACCTGCAGGCATGCAAGCTTCAGGGTTGAGATGTGTATAAGAGACAGCATCACCTCCGCAGGCGTAACGGGCATTGCGAGGCGTTCTCTCACCGCTTTGGCGGCGTTCAGCCCGGCTAACACCGCGCTTTCGATATTACCGATGCCCACGCTGTCCTGATTGGCGAAGTGAATATTCTCAAACGGCATGCTGGCCTGGTGAAACACGCCCTGAGCCTGTTGTCCGGCGACCGAGGTCACCAGACCCGCGGGCCAGAACCAGATTTGAATGTCTTCAATGATGTCGGCGTCAATGCCCATGTTGCCCAGCACTTTGGCGATTTCGACATAGGTCTGTTGCTGCAGCGCCGGGAACTCGTTCTGCGCGGTGCGACCTTGCCGCTCTGGGCGAGTGGTCGGTTTGAGCAGGGTTAATACCCCGGTTTCCGCGCTGAGGTGATTGTCCATCCAATTGGCGATCAGGCAGGTGCCGGCTTTGCTCCAATAGTAAGGTGTGCGGGTGTTTTCCGGCTCGATCAGATAGCCGCCGAACGAGTGTTCGAGGATCGGTTTTTTCAACAGCACGTTCGCCAGGTAGTAATCTTCATGGCGGATCTGACGGATCGCCGCCAATTGCTCTTTGGGCAGGGTGGGCAACCAGGTGGGGCACATGCGTTTTGGGGGCTGCCCAGATCACTTGTTTGGCTCTGATTTCGCTGGCCGCGCCATTGACGCTGAACCGGATCACCGTTTCGTCGCCGCCGGGCGTGATGCTTTGAAGCTGTACGCGGCTTTGCAGCCTGACGTTGTTTTGCTCCTCAAGATATTCGACCAGCCCGCGCCTGATGCTGCCGTTTACTGCCCGGCAGCGTGACCAGATTGCCGCGTAATACCCCACCAGGAGTGCATCCGACCAACGCCGGAAACTTTCCGAATATCAAGACATCCATGGCGCACGGTGTGCTGAACGCAATCTCTACCACCTTGCCCGAATTTTCTCGTGGC

**Sequencing result of sample2 lane(a)**

TAGCGGGGCGGTGGATCGACTTTTGCTGAGTTGAGGATCAGATCACGCATCTTCCCGACAACGCAGACCGTTCCGTGGCAAAGCAAAAGTTCAAAATCACCAACTGGTCCACCTACAACAAAGCTCTCATCAACCGTGGCGGGGATCCTCTAGAGTCGACCTGCAGGCATGCAAGCTTCAGGGTTGAGATGTGTATAAGAGACAGGCTCAACGCCATGAGCGAACAGAGCCTGTTTATGATCAAAAGCCGCGTCGCTCAGATCACCACCCCCTTCTCCGACGGCAGGCTGGAGCGCAAGGTTCACCTGGTGCAGCTTACGCACGATGAGCGCCATCTGTCGCCCTATGCCGATCTGCACCTGCACATGAACTGTCTGGCGTTCCTGCGCACGGTGAAAGCGCATCTCGACGTCGATCCGGCATTGCGGGAAAGACGCCGCGCGCTGATCGCCGCCTATCTCGACTCGCCTTCCGACGTGGTCAGCCAGCTTCCCAGTCTGCCCATGTCGGCCAACTACTTCTTCTGCCAGTTGAACCGAGTGATTGAAGAGCTGATCGAAACGGAGGGGTTCGATTTTACCGGCGTATATGACGTCGGCCGGTGCGGCATCTCCGCCGCGCGCAACGTCGCCAAAACGCGCCGCGGCTTTTCCGGCTGGTATGGCCGCGCCCTGATGGGCGACGCTCTGCTGGCCACCGGCTACCTGGCTTACACCAGCCCAAGCCACGTGATGGCGTTTATCGGCGACGGCGCTAAAGGGATCGTACCGGACATCCTGCCGGCTTTTATCGACAACATTCTTACCCATCCACAGTTGCTGAATAAAAGCATCACCGTCTTCTATTTATGCAACGGCGGGCTGTCCGTCATCAATACCTATCAGAACGCATTCTGTTCAACCGCACCTCACGGCAAATGCGGTTGGTGAACGTCGAACAGCCGGACGTCGAACAAACGGTCAATAACTTCCATATCCAGAGCAAAACGCTTACGCATTTCGACGAAGACGTCATTCGCCAGCGCTGACACGTCGCACCGACTCAATTTTGTCCTCGTGGTGCTGGGCACAACAATGAAAGGCGACGGTATTCTCCCCTGGCAT

**Sequencing result of sample2 lane(b)**

GGTGTAATCGACTTTTGCTGAGTTGAGGATCAGATCACGCATCTTCCCGACAACGCAGACCGTTCCGTGGCAAAGCAAAAGTTCAAAATCACCAACTGGTCCACCTACAACAAAGCTCTCATCAACCGTGGCGGGGATCCTCTAGAGTCGACCTGCAGGCATGCAAGCTTCAGGGTTGAGATGTGTATAAGAGACAGGCTCAACGCCATGAGCGAACAGAGCCTGTTTATGATCAAAAGCCGCGTCGCTCAGATCACCACCCCCTTCTCCGACGGCAGGCTGGAGCGCAAGGTTCACCTGGTGCAGCTTACGCACGATGAGCGCCATCTGTCGCCCTATGCCGATCTGCACCTGCACATGAACTGTCTGGCGTTCCTGCGCACGGTGAAAGCGCATCTCGACGTCGATCCGGCATTGCGGGAAAGACGCCGCGCGCTGATCGCCGCCTATCTCGACTCGCCTTCCGACGTGGTCAGCCAGCTTCCCAGTCTGCCCATGTCGGCCAACTACTTCTTCTGCCAGTTGAACCGAGTGATTGAAGAGCTGATCGAAACGGAGGGGTTCGATTTTACCGGCGTATATGACGTCGGCCGGTGCGGCATCTCCGCCGCGCGCAACGTCGCCAAAACGCGCCGCGGCTTTTCCGGCTGGTATGGCCGCGCCCTGATGGGCGACGCTCTGCTGGCCACCGGCTACCTGGCTTACACCAGCCCAAGCCACGTGATGGCGTTTATCGGCGACGGCGCTAAAAGGGATCGTACCGGACATCCTGCCGGTCTTTTATCGACAACATTCTTACCCATCCACAGTTGCTGAATAAAAGCATCACCGTCTTCTATTTATGCCACGGCGGGCTGTTCCGTCATCAATACCTATCAGGACCGCATTCTGTTCAACCGCACTTTAACGGCAAATGCGGTTGGTGAACGTCCGAACAGCCGGACGTCGAACAAACGGGTCAATACTTTCCATATCCAGAGCAAAACGCTTACGCATTTCCACTAAAGAAGTCCATCCGCCCAGTGGCTGAACACCCCGCCACCGAATTCATTCCGTCCTCGGTGTGTTGGA

**Sequencing result of sample2 lane(c)**

GGGGTTTTTATCGACTTTTGCTGAGTTGAGGATCAGATCACGCATCTTCCCGACAACGCAGACCGTTCCGTGGCAAAGCAAAAGTTCAAAATCACCAACTGGTCCACCTACAACAAAGCTCTCATCAACCGTGGCGGGGATCCTCTAGAGTCGACCTGCAGGCATGCAAGCTTCAGGGTTGAGATGTGTATAAGAGACAGGCTCAACGCCATGAGCGAACAGAGCCTGTTTATGATCAAAAGCCGCGTCGCTCAGATCACCACCCCCTTCTCCGACGGCAGGCTGGAGCGCAAGGTTCACCTGGTGCAGCTTACGCACGATGAGCGCCATCTGTCGCCCTATGCCGATCTGCACCTGCACATGAACTGTCTGGCGTTCCTGCGCACGGTGAAAGCGCATCTCGACGTCGATCCGGCATTGCGGGAAAGACGCCGCGCGCTGATCGCCGCCTATCTCGACTCGCCTTCCGACGTGGTCAGCCAGCTTCCCAGTCTGCCCATGTCGGCCAACTACTTCTTCTGCCAGTTGAACCGAGTGATTGAAGAGCTGATCGAAACGGAGGGGTTCGATTTTACCGGCGTATATGACGTCGGCCGGTGCGGCATCTCCGCCGCGCGCAACGTCGCCAAAACGCGCCGCGGCTTTTCCGGCTGGTATGGCCGCGCCCTGATGGGCGACGCTCTGCTGGCCACCGGCTACCTGGCTTACACCAGCCCAAGCCACGTGATGGCGTTTATCGGCGACGGCGCTAAAGGGATCGTACCGGACATCCTGCCGGCTTTTATCGACAACATTCTTACCCATCCACAGTTGCTGAATAAAGCATCACCGTCTTCTATTTATGCAACGGCGGGCTGTCCGTCATCAATACTTATCA

**Sequencing result of sample3 lane (a)**

GGGGGGCGGTGGATTCGACTTTTGCTGAGTTGAAGGATCAGATCACGCATCTTCCCGACAACGCAGACCGTTCCGTGGCAAAGCAAAAGTTCAAAATCACCAACTGGTCCACCTACAACAAAGCTCTCATCAACCGTGGCGGGGATCCTCTAGAGTCGACCTGCAGGCATGCAAGCTTCAGGGTTGAGATGTGTATAAGAGACAGCTTCGGTGCTGCGTGATTGCAACGGACGCCCACTTAACCCGCCCGCTTGTTCACAGTAATTCAGCCCTTGGATCAATTTTCGATCCAACGTGGTATTGGTGGCGATCACGCCATCAATATTATGCCGCACCAGGCTATCGGCAATTTGGATCAATTCTTCTTCAGAAAGATCCGGCGCGATCTTTACCGCCACCGGCACATATTTGTGATGGCGCGCATGTAATTCCTGCTGCTTATTTTTAATCGCAGCCAAAAGATCGTCCAACGCTTCGCCATACTGTAAGGATCGTAACCCCGGCGTATTCGGTGAAGAGATGTTAATCGCGATATATCCGGCATACGGATAAACTTTATCCATGCAGATCAAATAATCGTCTTTGCCCTGCTCGACCGGCGTGTCTTTATTCTTGCCGATATTGATCCCAAGAATGCCGCCGAAATGGGATTTCTTAACGTTTTCAACAAGATTATCCACGCCATGGTTATTGAAGCCCATGCGGTTAATCAGGCCTTCCGCCTCAACCACGCGGAACAAGCGCGGCTTGTCGTTGCCCGGTTGCGGACGCGGCGTCACGGTGCCGACTTcCACATGGCCGAAGCCCATCGTGCCGAACGCATCGATGCATTCGCCGTTTTTATCCAGACCGGCGGCCAATCCCAGTGGGTTTTTAAACGACAGTCCCATGCAGCTGACCGGTTTGGTCGGCACGGACTGACGGACAAGAAACGCTAATGGGGTGCCGGTAATACGGCTGAGCTGACGAAAGGTCACTTCGTGCGCGCGCTCCGGATCGA

**Sequencing result of sample3 lane (b)**

GGGGCCGGTGATTCGACTTTTGCTGAGTTGAGGTCAGATCACGCATCTTCCCGACAACGCAGACCGTTCCGTGGCAAAGCAAAAGTTCAAAATCACCAACTGGTCCACCTACAACAAAGCTCTCATCAACCGTGGCGGGGATCCTCTAGAGTCGACCTGCAGGCATGCAAGCTTCAGGGTTGAGATGTGTATAAGAGACAGCTTCGGTGCTGCGTGATTGCAACGGACGCCCACTTAACCCGCCCGCTTGTTCACAGTAATTCAGCCCTTGGATCAATTTTCGATCCAACGTGGTATTGGTGGCGATCACGCCATCAATATTATGCCGCACCAGGCTATCGGCAATTTGGATCAATTCTTCTTCAGAAAGATCCGGCGCGATCTTTACCGCCACCGGCACATATTTGTGATGGCGCGCATGTAATTCCTGCTGCTTATTTTTAATCGCAGCCAAAAGATCGTCCAACATCAATACAACCTA

**Sequencing result of sample 3 lane (c)**

GAGGCGGGCGGTTAATCGACTTTTGCTGAGTTGAGGATCAGATCACGCATCTTCCCGACAACGCAGACCGTTCCGTGGCAAAGCAAAAGTTCAAAATCACCAACTGGTCCACCTACAACAAAGCTCTCATCAACCGTGGCGGGGATCCTCTAGAGTCGACCTGCAGGCATGCAAGCTTCAGGGTTGAGATGTGTATAAGAGACAGCTTCGGTGCTGCGTGATTGCAACGGACGCCCACTTAACCCGCCCGCTTGTTCACAGTAATTCAGCCCTTGGATCAATTTTCGATCCAACGTGGTATTGGTGGCGATCACGCCATCAATATTATGCCGCACCAGGCTATCGGCAATTTGGATCAATTCTTCTTCAGAAAGATCCGGCGCGATCTTTACCGCCACCGGCACATATTTGTGATGGCGCGCATGTAATTCCTGCTGCTTATTTTTAATCGCAGCCAAAAGATCGTCCAACGCTTCGCCATACTGAAAGGATCGTAACCCCGGCGTATTCGGTGAAGAGATGTTAATCGCGATATATCCGGCATACGGATAAACTTTATCCATGCAGATCAAATAATCGTCTTTGCCCTGCTCGACCGGCGTGTCTTTATTCTTGCCGATATTGATCCCAAGAATGCCGCCGAAATGGGATTTCTTAACGTTTTCAACAAGATTATCCACGCCATGGTTATTGAAGCCCATGCGGTTAATCAGGCCTTCCGCCTCAACCACGCGGAACAAGCGCGGCTTGTCGTTGCCCGGTTGCGGACGCGGCGTCACGGTGCCGACTTCCACATGGCCGAAGCCCATCGTGCCGAACGCATCGATGCATTCGCCGTTTTTATCCAGACCGGCGGCCAATCCCAGTGGGTTTTTAACGACAGTCCCATGCAGCTGACCGGTTTGGTCGGCACGGACTGACGGACAGAAACGCTAATGGGGTGCCGGTAATACAGCTGAGCTGACGAAAGGTCACTTCGTGCGCGCGCTCCGGATCGAGC
